# Activation of the Nrf2/ARE Pathway Attenuates BDE-47-Induced Immunotoxicity in RAW264.7 Macrophages

**DOI:** 10.3390/biom16050674

**Published:** 2026-05-01

**Authors:** Qian Gao, Qingyuan Deng, Ziying Yang, Lili Wei, Hongmei Chen

**Affiliations:** 1 Key Laboratory of Xinjiang Phytomedicine Resources and Utilization, Ministry of Education, School of Pharmacy, Shihezi University, Shihezi 832000, China; 2 Department of Pharmacology, School of Pharmacy, Shihezi University, Shihezi 832000, China; 3 Heze Institute for Food and Drug Control, Heze 274000, China; 4 Key Laboratory of Xinjiang Endemic and Ethnic Diseases, Ministry of Education, Medical College, Shihezi University, Shihezi 832000, China

**Keywords:** BDE-47, Nrf2, macrophage, immunotoxicity, apoptosis, network toxicological analysis

## Abstract

Polybrominated diphenyl ethers (PBDEs), widely used as brominated flame retardants, are known to exert persistent adverse effects on the immune systems of humans and other organisms. Previous studies have demonstrated that 2,2′,4,4′-tetrabromodiphenyl ether (BDE-47), a prevalent congener, induces apoptosis, impairs phagocytic function, and triggers aberrant immune-inflammatory reactions in RAW264.7 macrophages via the induction of elevated intracellular reactive oxygen species (ROS). However, the underlying regulatory mechanism remains unclear. The nuclear factor erythroid 2-related factor 2/antioxidant response element (Nrf2/ARE) signaling pathway is a key cellular defense system against oxidative stress. In this study, we investigated the role of the Nrf2/ARE pathway in BDE-47-induced macrophage immunotoxicity. Network toxicology analysis identified Nrf2 as a hub gene within the BDE-47-associated immunotoxicity network. Molecular docking and molecular dynamics simulations suggested a potential interaction between BDE-47 and the Keap1-Nrf2 complex, with moderate binding affinity. Experimental studies in RAW264.7 cells showed that BDE-47 exposure activated the Nrf2/ARE pathway, as evidenced by Nrf2 nuclear translocation and the differential upregulation of downstream genes (GCLC, GCLM, HO-1, NQO1, SOD1, and CAT). Importantly, Nrf2 knockdown via lentiviral shRNA or pharmacological inhibition with brusatol significantly exacerbated BDE-47-induced apoptosis and immune dysfunction, including enhanced pro-inflammatory cytokine production and impaired phagocytosis. These results demonstrate that Nrf2/ARE pathway activation represents an adaptive antioxidant response and contributes to limiting BDE-47-induced cytotoxicity and immune impairment in macrophages.

## 1. Introduction

Polybrominated diphenyl ethers (PBDEs) are a class of brominated flame retardant widely used in electronics, textiles, furniture, and building materials [1,2]. These compounds are not chemically bound to the substrate and can be continuously released into the environment. Due to their lipophilicity, PBDEs readily bioaccumulate in organisms and biomagnify along food chains, posing a potential threat to human health [3]. Highly brominated PBDEs are also susceptible to photodegradation, leading to the formation of lower-brominated homologues [4]. Among these, 2,2′,4,4′-tetrabromodiphenyl ether (BDE-47) is one of the most frequently detected low-brominated PBDE congeners in environmental samples and human tissues [5]. Previous studies have demonstrated that BDE-47 exerts multiple adverse effects on the nervous, endocrine and developmental systems. Thus, BDE-47 is regarded as a key indicator for assessing the health risks associated with PBDE exposure [6].

The immune system serves as the body’s primary defense mechanism and is highly sensitive to external environmental factors. Recent studies have revealed the emerging potential of BDE-47 [7]. Current evidence demonstrates that BDE-47 exposure impairs immune organ function in organisms such as rainbow trout, thereby reducing their resistance to pathogens [8]. In addition, BDE-47 interferes with both innate and adaptive immune responses, leading to decreased immune competence in mice [7]. These immunotoxic effects are manifested by abnormal inflammatory responses [9,10], dysregulation of immune function [11], immune cell apoptosis [12], and dysfunction of immune organs [13]. In vitro investigations further demonstrate that BDE-47 impairs the normal functions of diverse immune cell types, including human THP-1 monocytes and fish neutrophils [14,15]. Notably, macrophages play a crucial role in host defense by mediating phagocytosis and immune regulation. Previous studies have shown that BDE-47 induces oxidative stress and significantly alters the inflammatory cytokine secretion in macrophages from different species, thereby compromising immune homeostasis [16,17,18,19]. However, the precise molecular mechanisms underlying BDE-47-induced macrophage dysfunction remain incompletely understood. A systematic investigation of these mechanisms is therefore essential for a comprehensive assessment of its immunotoxic effects.

Macrophages play a critical role in defending against inflammation and damage caused by external pollutants, in part through oxidative bursts involving the production of reactive oxygen species (ROS) [20,21]. However, excessive ROS levels can lead to macromolecular damage and ultimately induce cell death [22]. Our previous study demonstrated that BDE-47 impairs immune function in RAW264.7 macrophages by inducing oxidative stress through elevated ROS levels [23]. Consistent with this, PBDEs have also been reported to promote ROS generation in various organisms and immune cells, leading to oxidative stress and subsequent immune dysfunction [24,25,26,27]. Although these findings establish ROS-mediated oxidative stress as a key event, the core regulatory genes and precise signaling pathways underlying this process remain unclear. The nuclear factor erythroid 2-related factor 2 (Nrf2)/antioxidant response element (ARE) signaling pathway represents a major cellular defense mechanism against oxidative stress and xenobiotic-induced toxicity. Activation of this pathway induces the expression of a broad range of antioxidant and detoxifying enzymes, thereby enhancing cellular resistance to oxidative damage [28,29]. Under physiological conditions, Nrf2 is predominantly retained in the cytoplasm through interaction with Kelch-like ECH-associated protein 1 (Keap1). Upon exposure to oxidative or electrophilic stress, this interaction is disrupted, allowing Nrf2 to translocate into the nucleus and activate the transcription of cytoprotective genes, including those encoding phase II detoxification enzymes and antioxidant enzymes such as NAD(P)H quinone oxidoreductase 1 (NQO1) and heme oxygenase-1 (HO-1). These enzymes facilitate the elimination of excess reactive species, maintain intracellular redox homeostasis, and exert cytoprotective effects [29,30,31]. The Nrf2/ARE pathway is increasingly recognized for its role in mediating cellular defense against environmental pollutants, including heavy metals such as mercury and cadmium [32]. Moreover, compounds such as tert butylhydroquinone (tBHQ) and sulforaphane have been shown to activate the Nrf2/ARE pathway, enhancing antioxidant gene expression and alleviating BDE-47-induced oxidative stress and inflammation in HTR 8/SVneo cells [33]. These findings indicate that the Nrf2/ARE signaling pathway may play a crucial role in the immune response to PBDE-induced oxidative stress and immune dysfunction.

At present, humans remain widely exposed to PBDEs in the environment, which may lead to varying degrees of oxidative damage and immune toxicity [34,35,36]. Therefore, elucidating the molecular mechanisms underlying BDE-47-induced immunotoxicity is of significant scientific importance. With the advancement of systems biology, network toxicology has emerged as a powerful approach for deciphering complex toxicological mechanisms. Compared with traditional methods, network-based analysis enables more efficient and comprehensive evaluation of toxicity from a systems-level perspective, facilitating the identification of key regulatory targets and pathways [37]. In parallel, molecular docking coupled with dynamics simulation provides an effective strategy for predicting ligand–receptor interactions, offering valuable insights into the potential interactions between pollutants and biological macromolecules [38]. This study integrates network toxicology analysis with molecular docking and molecular dynamics simulation to screen and validate pivotal genes involved in BDE-47-induced immunotoxicity. Furthermore, molecular biology techniques and gene silencing approaches ware applied to investigated the regulatory role of the potential target Nrf2 in BDE-47-induced RAW264.7 cell toxicity. The study aim is to elucidate the potential immunotoxicity mechanisms of PBDEs and provide a mechanistic foundation for future risk assessment and intervention strategies.

## 2. Materials and Methods

### 2.1. Chemicals and Reagents

BDE-47 Standard (Purity ≥ 99.9%) was purchased from Accustandard (New Haven, CT, USA). Brusatol (SML1868), 2′,7′-dichlorodihydrofluorescein diacetate (DCFH-DA, 35845), 3-(4,5-dimethylthiazol-2-yl)-2,5-diphenyltetrazolium bromide (MTT, 475989), and dimethyl sulfoxide (DMSO, D8418) were obtained from Sigma-Aldrich (St. Louis, MO, USA). The Nrf2 antibody (16396-1-AP) was acquired from Proteintech (Wuhan, China). Horseradish peroxidase (HRP)-conjugated secondary antibody (D110011) was obtained from Sangon Biotech (Shanghai, China). The apoptosis detection kit was acquired from MultiSciences (Hangzhou, China). Escherichia coli (strain K-12) was procured from Invitrogen (Carlsbad, CA, USA). DAPI solution (C0065), a nuclear and cytoplasmic protein extraction kit (P0027), enhanced chemiluminescence (ECL) reagent (P0018AS), and ELISA kits of IL-6 (PI326), IL-1β (PI301), and TNF-α (PT512) were procured from Beyotime Biotechnology (Shanghai, China). Inducible nitric oxide synthase (iNOS) antibodies were procured from eBioscience (San Diego, CA, USA). Real-time PCR reagents were procured from Takara Biomedical Technology Co., Ltd. (Beijing, China).

### 2.2. Acquisition of Potential Targets for BDE-47-Induced Immune Dysfunction

The Comparative Toxicogenomics Database (CTD, http://ctdbase.org/, accessed on 15 March 2023) provides comprehensive toxicological data [39]. Potential toxicity targets of BDE-47 were retrieved from the CTD database using “BDE-47” as a keyword. Swiss Target Prediction (http://www.swisstargetprediction.ch/, accessed on 15 March 2023) [40] and PharmMapper Server (http://www.lilab-ecust.cn/pharmmapper/, accessed on 15 March 2023) [41] were employed to predict molecular targets based on compound structural similarity and pharmacophore matching, respectively. The three-dimensional (3D) structure of BDE-47 was downloaded from PubChem (https://pubchem.ncbi.nlm.nih.gov/, accessed on 16 March 2023) prior to its submission to the aforementioned dual databases. All predicted targets from the three databases were standardized to official gene symbols using the UniProt database (http://www.uniprot.org/, accessed on 15 March 2023).

Immunotoxicity-related targets were identified by searching the GeneCards database (https://www.genecards.org/, accessed on 16 March 2023) using the keywords “immunotoxicity”, “immune injury” and “immune damage”. The common targets derived from the three search results were considered core immunotoxicity-related genes. The intersection between these core immunotoxicity targets and the potential targets of BDE-47 was then determined, yielding the candidate targets associated with BDE-47-induced immunotoxicity.

### 2.3. Network Toxicological Analysis of BDE-47-Induced Immunotoxicity

To investigate interactions among targets associated with BDE-47 immunotoxicity, the integrated genes were uploaded to the STRING database to construct a protein–protein interaction (PPI) network. The resulting network data was exported as TSV files and subsequently imported into Cytoscape (version 3.7.2) for visualization and topological analysis. Node degree was calculated to quantify connectivity, with node size and color encoding connection numbers and binding scores, respectively. The MCODE algorithm was applied for module detection, followed by CytoHubba analysis to identify hub genes within subnetworks.

The subnetworks identified from the network analysis were further subjected to functional enrichment analysis using the Metascape database (http://metascape.org/, accessed on 17 March 2023). Gene Ontology (GO) terms and Kyoto Encyclopedia of Genes and Genomes (KEGG) pathways were analyzed, and the top enriched results were visualized using Hiplot (http://dev.hiplot-academic.com, accessed on 17 March 2023), a cloud-based platform for biomedical data visualization.

### 2.4. Molecular Docking and Dynamics Simulation Verified the Binding of Potential Regulatory Gene Nrf2 to BDE-47

Based on the network analysis results, Nrf2 was identified as a key candidate gene associated with oxidative stress and was selected for further computational analysis. The crystal structure of the Nrf2/Keap1 complex (PDBID: 6qmj) was obtained from the Protein Data Bank (PDB, http://www.rcsb.org/, accessed on 3 April 2023) and used as the acceptor. Water molecules and protoligands were removed using PyMOL2.3.0 to prepare the protein for docking. The three-dimensional structure of BDE-47 was obtained from the PubChem database as the ligand. Molecular docking was performed using AutoDock Vina v.1.2.0 with a semi-flexible docking protocol. The exhaustiveness parameter was set to 8, and a maximum of 9 conformations were output. The docking results were visualized using the PyMOL2.3.0 software and Discovery Studio 2020.

To further explore the stability of the predicted binding mode, molecular dynamics (MD) simulations were carried out using Amber 22 (San Francisco, CA, USA) based on the optimal docking conformation. The ff14SB force field was applied to define system parameters. The system was solvated with the TIP3P water model and neutralized by adding counterions. After energy minimization, the system was heated from 0 K to 300 K over 500 ps under position restraints. Subsequently, equilibration was performed under the canonical (NVT) ensemble at 300 K, followed by further equilibrium in the isothermal isobaric system (NPT) ensemble. Finally, a 20 ns production MD simulation was executed under periodic boundary conditions with constant temperature and pressure maintained. All covalent bonds involving hydrogen were rigidly constrained via the SHAKE algorithm.

### 2.5. Cell Culture, Construction of Nrf2 Low-Expression Cell Models and Experiment Design

RAW264.7 cells were acquired from the Shanghai Institute of Cell Biology, Shanghai, China. We used Dulbecco’s modified Eagle’s medium (DMEM) containing 10% fetal bovine serum (FBS) and 1% penicillin–streptomycin at 37 °C in a humidified incubator at 5% CO_2_. Cells were seeded in cell culture plates at a density of 1 × 10^5^/mL and allowed to adhere for 12 h. Subsequently, cells were treated with BDE-47 at varying concentrations (5, 10, 20, and 40 μM) for 24 h. A blank control (CTR) and a DMSO solvent control (DMSO) were set at the same time.

For Nrf2 knockdown, RAW264.7 cells were infected with lentiviral vectors encoding short hairpin RNA targeting Nrf2 (shNrf2, Shanghai GenePharma Co., Ltd., Shanghai, China). To determine the optimal multiplicity of infection (MOI), preliminary experiments were conducted using MOIs of 1, 10, and 100, and transduction efficiency was evaluated by green fluorescent protein (GFP) expression and cell viability. Based on these optimization experiments, an MOI of 10 was selected to achieve efficient gene silencing while minimizing cytotoxicity. Cells were transduced with shNrf2 or control lentivirus and selected with puromycin (3 μg/mL) for 7–14 days to establish stable Nrf2-knockdown cell lines. The study comprised distinct experimental arms, including the control group (CTR, RAW264.7 cells), the BDE-47 group (RAW264.7 cells treated with 20 μM BDE-47), the negative control group (NC, RAW264.7 cells stably expressing scramble shRNA), the shNrf2 group (SN, RAW264.7 cells with Nrf2 knockdown), the BDE-47-treated NC cell group, and the BDE-47-treated SN cell group.

To complement the genetic approach, RAW264.7 cells were pretreated with the Nrf2 inhibitor brusatol (100 nM) for 24 h prior to BDE-47 exposure. Four experimental groups of CTR, BDE-47, Brusatol, and Brusatol associated with BDE-47 were set up. BDE-47 treatment concentration and duration were the same as above.

### 2.6. Western Blot Analysis

Following treatment, nuclear and cytoplasmic proteins were extracted using commercial kits and quantified by bicinchoninic acid (BCA) assay. Equal amounts of proteins were separated by sodium dodecyl sulfate–polyacrylamide gel electrophoresis (SDS-PAGE) and transferred to polyvinylidene fluoride (PVDF) membranes. Membranes were blocked with 5% skim milk for 2 h at room temperature, and incubated overnight at 4°C with primary antibodies against Nrf2 (1:1000), β-actin (1:1000), and Histone H3 (1:2000). After five TBST washes (5 min each), membranes were incubated with HRP-conjugated secondary antibody (1:10,000) for 1 h at room temperature. Protein bands were visualized via chemiluminescence (ECL). Original figures can be found in Supplementary Materials.

### 2.7. Immunofluorescence Staining

RAW264.7 cells were seeded onto sterile glass coverslips and allowed to attach for 2 h in a 5% CO_2_ incubator before being treated with varying concentrations of BDE-47 for 24 h. After treatment, cells were then fixed and permeabilized with acetone, blocked with 5% bovine serum albumin (BSA) in tris-buffered saline (TBS) for 2 h, and incubated with primary antibody against Nrf2 overnight at 4 °C. After washing with TBS, the cells were incubated with a fluorescein isothiocyanate (FITC)-conjugated secondary antibody for 30 min at room temperature. Nuclei were counterstained with 4′,6-diamidino-2-phenylindole (DAPI) (1 μg/mL). Fluorescent images were acquired on a Zeiss microscope (Oberkochen, Germany) and processed using Axio Vision 4.8 software.

### 2.8. Quantitative Real-Time PCR (qRT-PCR)

After treatment, total RNA was isolated from harvested cells, with concentration and purity assessed by spectrophotometry. First-strand cDNA was synthesized using a reverse transcription kit per the manufacturer’s protocol. Quantitative real-time PCR was performed under the following thermal conditions: initial denaturation at 95 °C for 3 min, followed by 30 amplification cycles (95 °C for 10 s, 55 °C for 30 s). Target gene expression was normalized to GAPDH and calculated by the 2^−ΔΔCt^ method. Primer sequences (Sangon Biotech, Shanghai, China) are provided in Table 1.

### 2.9. Transmission Electron Microscopy (TEM)

Treated cells were initially fixed with 2.5% glutaraldehyde at 4 °C for 2 h, then post-fixed with 1% osmium tetroxide at 37 °C for 2 h. Subsequent processing included PBS washing, progressive ethanol dehydration, acetone permeation, and resin polymerization. Ultrathin sections (60–80 nm) were prepared and double-stained with uranyl acetate and lead citrate. Cellular ultrastructure was observed using a transmission electron microscope.

### 2.10. Cell Viability Assay

The MTT assay was used to detect the effect of low Nrf2 expression on BDE-47-induced cell viability in RAW264.7 cells. Cells were seeded in 96-well plates at a density of 1 × 10^4^ cells per well, with five replicate wells per group. After a 24 h incubation period, 10 μL of MTT solution was added to incubate at a temperature of 37 °C for 4 h. Thereafter, the supernatant was removed, and dimethyl sulfoxide (DMSO) was introduced with vigorous shaking to fully solubilize the formazan crystals. Absorbance was measured at 490 nm using a microplate reader, and cell viability was calculated accordingly.

### 2.11. Reactive Oxygen Species Level Detection

Intracellular ROS levels were measured using 2′,7′-dichlorodihydrofluorescein diacetate (DCFH-DA). Cells were incubated with DCFH-DA at 37 °C for 20 min in the dark. The quantification of reactive oxygen species (ROS) fluorescence intensity for each experimental cohort was performed utilizing a Thermo Fisher multifunctional microplate reader (Waltham, MA, USA), employing an excitation wavelength of 488 nm and an emission wavelength of 525 nm. The relative ROS levels were expressed as fluorescence intensity compared with the control group.

### 2.12. Flow Cytometric Analysis: Apoptosis, Phagocytosis and iNOS Expression

For apoptosis analysis, cells were collected, washed twice with cold phosphate-buffered saline (PBS), and resuspended in binding buffer. Cells were then stained with Annexin V-FITC and propidium iodide (PI) for 20 min at room temperature in the dark. Apoptotic cells were analyzed by flow cytometry.

For phagocytosis assessment, cells were cultured in serum-free medium containing fluorescein isothiocyanate (FITC)-labeled Escherichia coli (K-12 strain) biotinylates at a ratio of 1:30. After incubation for 1 h at 37 °C, cells were harvested and washed three times with PBS to remove non-internalized particles. Phagocytic activity was then quantified by flow cytometry.

Macrophage M1-type marker iNOS expression levels were measured through flow cytometry [42,43]. Cells were fixed with 4% paraformaldehyde for 20 min at room temperature, washed twice with PBS, and permeabilized using a commercial permeabilization buffer. Subsequently, cells were incubated with an allophycocyanin (APC)-conjugated anti-iNOS antibody for 30 min at 4 °C in the dark. After washing, samples were analyzed by flow cytometry.

### 2.13. Cytokine and Nitric Oxide Detection

The supernatants were clarified through centrifugation and subsequently analyzed for nitric oxide (NO) using the Griess method. Additionally, pro-inflammatory cytokines IL-6, IL-1β, and TNF-α were quantified utilizing commercially available ELISA kits, following the specific protocols provided by the manufacturers.

### 2.14. Statistical Analysis

All experiments were performed with at least three independent biological replicates. Data are presented as mean ± standard error of the mean (SEM). Statistical analyses were conducted using SPSS software (version 26.0). Differences among groups were evaluated by one-way analysis of variance (ANOVA), followed by Tukey’s post hoc test for multiple comparisons. A *p*-value < 0.05 was considered statistically significant. Graphs were generated using GraphPad Prism (version 8.4).

## 3. Results

### 3.1. Network Toxicology Predicts Nrf2 as a Key Candidate Gene in BDE-47-Induced Immunotoxicity

**Figure 1:**
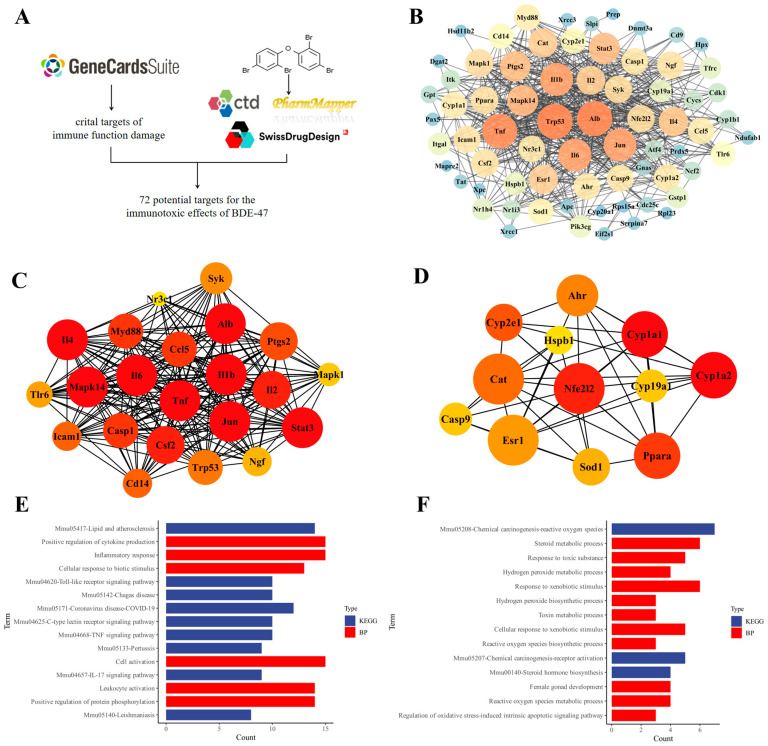
Network toxicology analysis of BDE-47 immunotoxicity. (**A**) The workflow for obtaining the intersection target of BDE-47 and immune damage. (**B**) Protein–protein interaction (PPI) network of potential immunotoxicity targets of BDE-47. (**C**,**D**) Two major subnetworks identified through MCODE modular analysis. (**E**,**F**) Gene ontology (GO) enrichment and Kyoto Encyclopedia of Genes and Genomes (KEGG) pathway analysis of genes in subnetwork 1 and subnetwork 2.

A total of 139 targets associated with immune dysfunction were identified from the GeneCards database. These targets were intersected with potential BDE-47 targets obtained from the CTD, SwissTarget Prediction, and PharmMapper databases, producing 72 common targets (Figure 1A). The overlapping targets were subsequently imported into the STRING platform to construct a protein–protein interaction (PPI) network (Figure 1B). Modular analysis of PPI network using the MCODE plugin in Cytoscape identified two distinct subnetworks (Figure 1C,D). Functional enrichment analysis of each subnetwork was performed using Metascape (Figure 1E,F). Genes in subnetwork 1 were primarily involved in biological processes such as positive regulation of cytokine production, inflammatory response, and cellular response to biotic stimulus. KEGG pathway analysis associated this subnetwork with lipid and atherosclerosis and Toll-like receptor signaling. In contrast, genes in subnetwork 2 were enriched in oxidative stress-related processes, including hydrogen peroxide metabolic process, response to exogenous stimuli, and regulation of intrinsic apoptotic signaling pathway. Corresponding KEGG pathways included chemical carcinogenesis via reactive oxygen species and related pathways.

Notably, our previous findings demonstrated that BDE-47-induced immunotoxicity in RAW264.7 cells is closely associated with ROS accumulation and oxidative stress. The enrichment profile of subnetwork 2 aligns with this observation. Based on network topology analysis, including degree and betweenness centrality, Nrf2 was identified as a highly connected node within this oxidative stress-associated subnetwork. Importantly, the prioritization of Nrf2 was not solely based on its known biological relevance, but also on its prominent topological position within the network and its direct association with ROS-related pathways. These findings support Nrf2 as a biologically relevant candidate.

### 3.2. Molecular Docking and Dynamics Simulations Suggest a Potential Interaction Between BDE-47 and the Keap1-Nrf2 Complex

**Figure 2:**
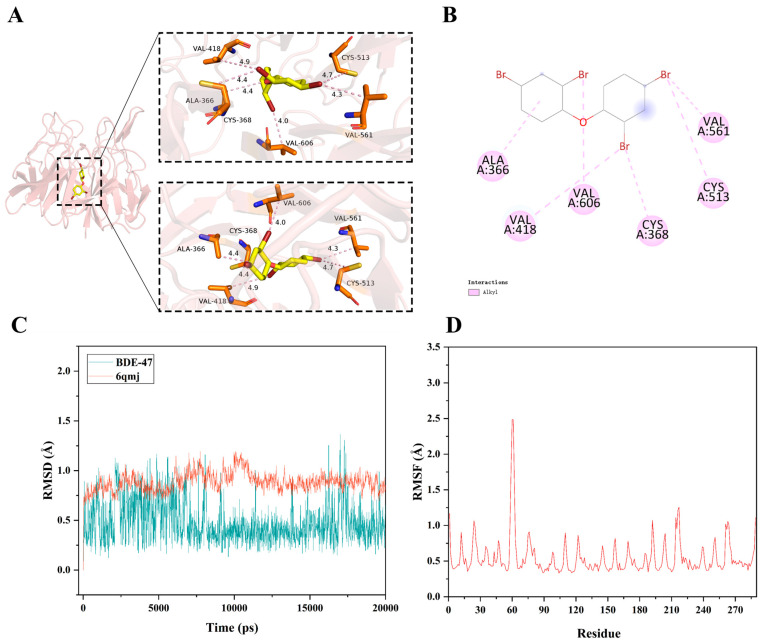
Molecular docking and dynamic simulation of BDE-47 with the Nrf2–Keap1 protein binding domain. (**A**) Three-dimensional structural diagram of molecular docking. (**B**) Two-dimensional structural diagram of molecular docking. (**C**) RMSD analysis of the molecular dynamics simulation. (**D**) RMSF analysis of the molecular dynamics simulation.

AutoDock was employed for molecular docking simulations to explore the potential binding mode of BDE-47 with the Keap1–Nrf2 complex. The calculated binding energy was −6.7 kJ/mol, suggesting a moderate interaction (values below −7 kJ/mol are generally indicative of robust ligand–receptor interactions). BDE-47 was located within a hydrophobic cavity formed by residues VAL-418, VAL561, VAL-606, ALA-366, CYS-361, and CYS-561, primarily through alkyl interactions (Figure 2A,B).

To further assess binding stability, molecular dynamics simulations were conducted. The root-mean-square deviation (RMSD) of the Nrf2/Keap1 complex and BDE-47 remained below 1.5 Å throughout the simulation, suggesting that the overall structure of the complex was relatively stable (Figure 2C). Root-mean-square fluctuation (RMSF) analysis revealed limited flexibility across residues, except for the terminus regions and residues 58–63, indicating relatively constrained conformational dynamics (Figure 2D). Taken together, these results suggest that BDE-47 may interact with the Keap1–Nrf2 interface and form a relatively stable complex under simulated conditions. However, this interaction should be interpreted as a potential association requiring further experimental validation.

### 3.3. BDE-47 Activates the Nrf2 Signaling Pathway and Induces Antioxidant Gene Expression in RAW264.7 Cells

**Figure 3:**
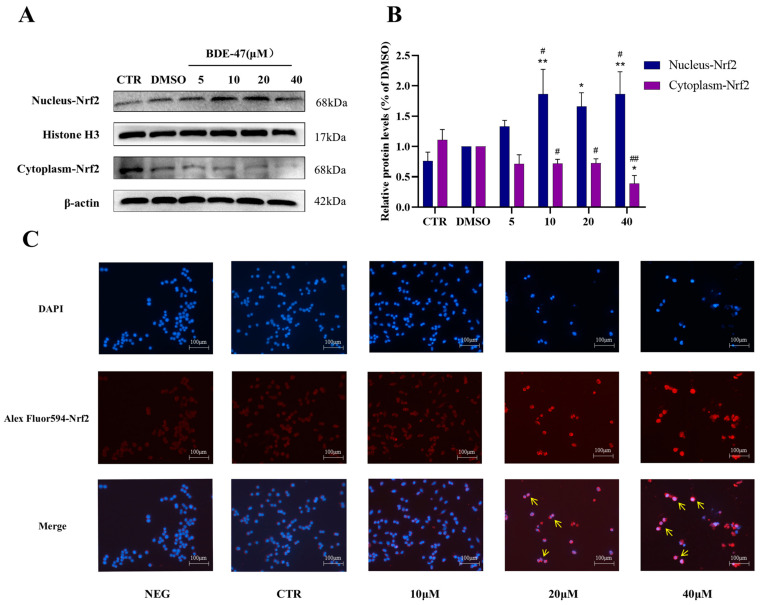
Effects of BDE-47 on Nrf2 expression and subcellular localization in RAW 264.7 cells. (**A**) Representative Western blot images showing nuclear and cytoplasmic Nrf2 protein levels. (**B**) Quantitative analysis of Nrf2 of protein expression. (**C**) Immunofluorescence staining of Nrf2 (red) and nuclei (DAPI, blue). The yellow arrows indicate nucleus Nrf2. Data are presented as mean ± SEM from three independent experiments (*n* = 3). Compared to the CTR group, * represents *p* < 0.05 and ** represents *p* < 0.01. Compared to the DMSO group, # represents *p* < 0.05 and ## represents *p* < 0.01.

Western blot analysis revealed no significant difference in Nrf2 expression between the DMSO control and the CTR. Compared with the DMSO group, BDE-47 treatment induced a concentration-dependent increase in nuclear Nrf2 expression from 10 to 40 μM, with the highest level observed at 40 μM. In contrast, cytoplasmic Nrf2 levels decreased progressively over the same concentration range, reaching a minimum at 40 μM (Figure 3A,B).

Immunofluorescence staining confirmed nuclear translocation of Nrf2 in RAW264.7 cells treated with BDE-47. In control cells, Nrf2 fluorescence (red) was predominantly localized in the cytoplasm, with minimal nuclear localization. Following BDE-47 treatment, Nrf2 fluorescence intensity increased and progressively accumulated in the nucleus, colocalizing with DAPI-stained nuclei (blue). This shift was evident across increasing concentrations of BDE-47, demonstrating concentration-dependent nuclear translocation of Nrf2 (Figure 3C).

**Figure 4:**
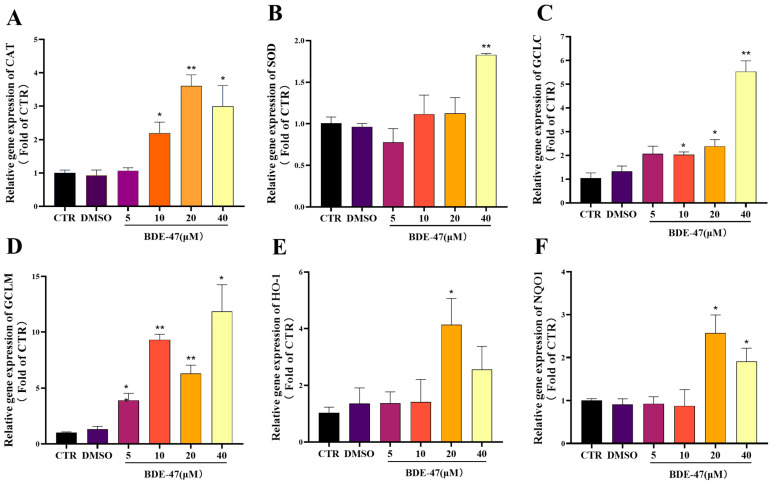
Effect of BDE-47 on the mRNA expression of antioxidant genes and Nrf2-related genes in RAW 264.7 cells. The mRNA expression of CAT (**A**), SOD1 (**B**), GCLC (**C**), GCLM (**D**), HO-1 (**E**), and NQO1 (**F**) was determined by qPCR. Data are presented as mean ± SEM from three independent experiments (*n* = 3). Compared with the CTR group, * represents *p* < 0.05, ** represents *p* < 0.01.

The mRNA expression of antioxidant genes and several Nrf2-regulated genes was measured with quantitative real-time PCR (qPCR). BDE-47 exposure increased the expression of catalase (CAT), superoxide dismutase 1 (SOD1), glutamate-cysteine ligase catalytic subunit (GCLC), glutamate-cysteine ligase modifier subunit (GCLM), HO-1, and NQO1, although the magnitude of induction varied among genes (Figure 4). SOD1 and GCLC expression showed a concentration-dependent increase, reaching peak levels at 40 μM BDE-47. GCLM expression was significantly upregulated at concentrations above 5 μM and also peaked at 40 μM. In contrast, CAT, HO-1, and NQO1 expression peaked at 20 μM BDE-47 and decreased at 40 μM, exhibiting a trend similar to that observed for nuclear Nrf2 protein levels.

### 3.4. Nrf2 Suppression Amplifies BDE-47 Cytotoxicity in RAW264.7 Macrophages

#### 3.4.1. Establishment and Validation of Nrf2-Knockdown Macrophage Model

**Figure 5:**
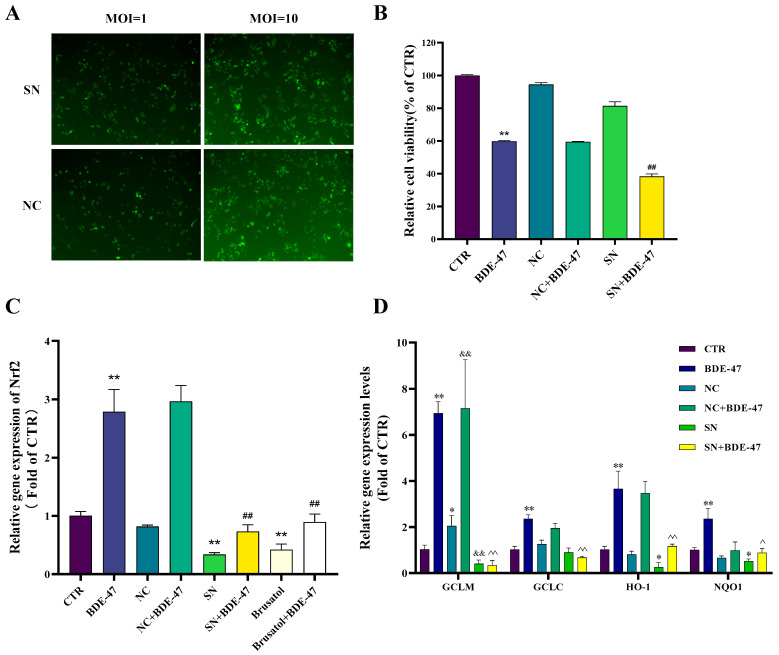
Validation of the Nrf2-suppressed RAW264.7 cell model. (**A**) GFP fluorescence images showing transduction efficiency of shRNA lentivirus at different multiplicities of infection (MOIs, 10×). (**B**) Cell viability. (**C**) Effect of lentiviral transfection and brusatol on Nrf2 gene expression. (**D**) Effect of lentiviral transfection on the expression of Nrf2 downstream target genes. Data are presented as mean ± SEM from three independent experiments (*n* = 3). Compared with the CTR group, * represents *p* < 0.05 and ** represents *p* < 0.01. Compared with BDE-47, ## represents *p* < 0.01. Compared with NC, && represents *p* < 0.01. Compared with NC+BDE-47, ^ represents *p* < 0.05 and ^^ represents *p* < 0.01.

A stable Nrf2-knockdown cell model was established using lentiviral delivery of Nrf2-targeting shRNA. Based on preliminary optimization, an MOI of 10 was selected to achieve efficient transduction while maintaining normal cell morphology (Figure 5A). Cell viability was not affected by shRNA transduction alone (NC or SN vs. CTR). However, BDE-47 exposure reduced cell viability in all treated groups, with the greatest reduction observed in BDE-47-treated SN cells (*p* < 0.01 vs. BDE-47-treated NC cells, Figure 5B). QRT-PCR analysis confirmed effective suppression of Nrf2 expression under both genetic and pharmacological interventions. Lentivirus-mediated shRNA knockdown (SN group) significantly reduced Nrf2 mRNA levels compared to the non-targeting control (NC) (*p* < 0.01). Similarly, treatment with the Nrf2 inhibitor brusatol markedly decreased Nrf2 expression (*p* < 0.01, Figure 5C). BDE-47 exposure significantly increased Nrf2 gene expression level in RAW264.7 cells. However, this induction was markedly attenuated in SN cells exposed to BDE-47. A comparable dampening effect was observed in cells pretreated with brusatol before BDE-47 exposure (Figure 5C). Consistent with suppression of the Nrf2 pathway, the expression of downstream targets, including GCLC, GCLM, HO-1, and NQO1, was significantly reduced in SN cells (Figure 5D).

#### 3.4.2. Nrf2 Suppression Exacerbates BDE-47-Induced Ultrastructural Damage, Oxidative Stress, and Apoptosis in RAW264.7 Cells

**Figure 6:**
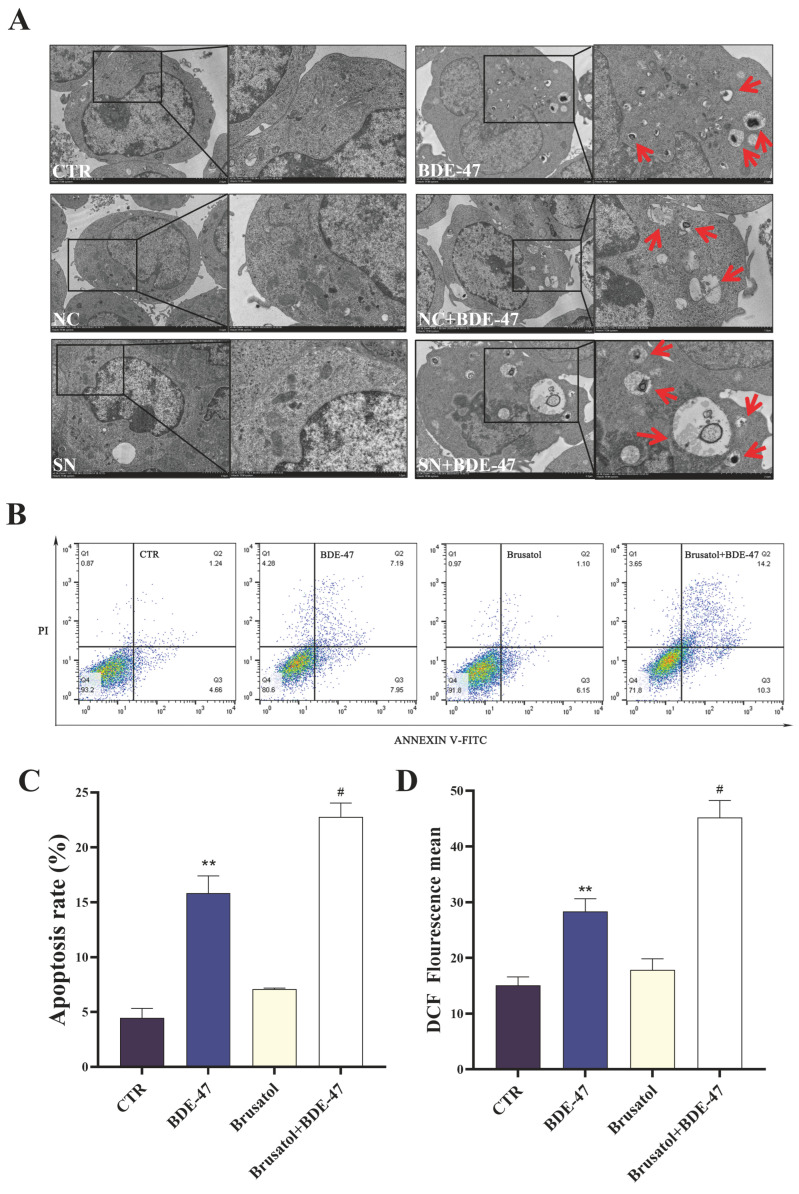
Effects of Nrf2 inhibition on cellular ultrastructure, apoptosis and ROS levels in BDE-47-treated RAW264.7 cells. (**A**) Representative transmission electron microscopy images showing ultrastructural changes. Red arrows indicate intracellular vacuoles and phagocytic structures. (**B**,**C**) Flow cytometry analysis of apoptosis, including representative dot plots and quantitative results. (**D**) Quantification of intracellular ROS levels under the same treatment conditions. Data are presented as mean ± SEM from three independent experiments (*n* = 3). Compared to the CTR group, ** represents *p* < 0.01. Compared to BDE-47, # represents *p* < 0.05.

Transmission electron microscopy (TEM) was used to examine cellular ultrastructure. In control cells, cellular architecture was intact. Mitochondria were predominantly rod-shaped or ellipsoidal with continuous outer membranes, and cristae were loosely organized. The endoplasmic reticulum was irregularly distributed, and no obvious apoptotic vesicles were observed. In BDE-47-treated cells, overall cellular morphology was largely preserved. However, phagocytic vesicles and apoptotic bodies were observed (indicated by red arrows). Cells transduced with non-targeting shRNA (NC) exhibited ultrastructural features comparable to those of control cells. In contrast, Nrf2-knockdown (SN) cells displayed blurred plasma membrane boundaries and reduced organelle integrity. Upon BDE-47 exposure, NC cells showed cytoplasmic vacuolation similar to that observed in BDE-47-treated cells. Notably, SN cells treated with BDE-47 exhibited more pronounced vacuolization, with enlarged vacuoles containing electron-dense materials (Figure 6A).

To further evaluate cellular damage, apoptosis and intracellular ROS level were measured. BDE-47 treatment significantly increased the apoptotic rate of RAW264.7 cells. Brusatol alone induced a slight, non-significant increase in apoptosis, whereas co-treatment with BDE-47 significantly enhanced the apoptotic rate compared with either treatment alone (*p* < 0.05, Figure 6B,C). Consistent with these findings, intracellular ROS levels were significantly elevated following BDE-47 exposure, reaching approximately 1.94-fold those of the control group. Brusatol alone did not significantly alter intracellular ROS levels. However, co-treatment with BDE-47 further elevated ROS accumulation compared with BDE-47 treatment alone (*p* < 0.01, Figure 6D).

### 3.5. Nrf2 Inhibition Exacerbates BDE-47-Induced Dysregulation of Macrophage Immune Functions

**Figure 7:**
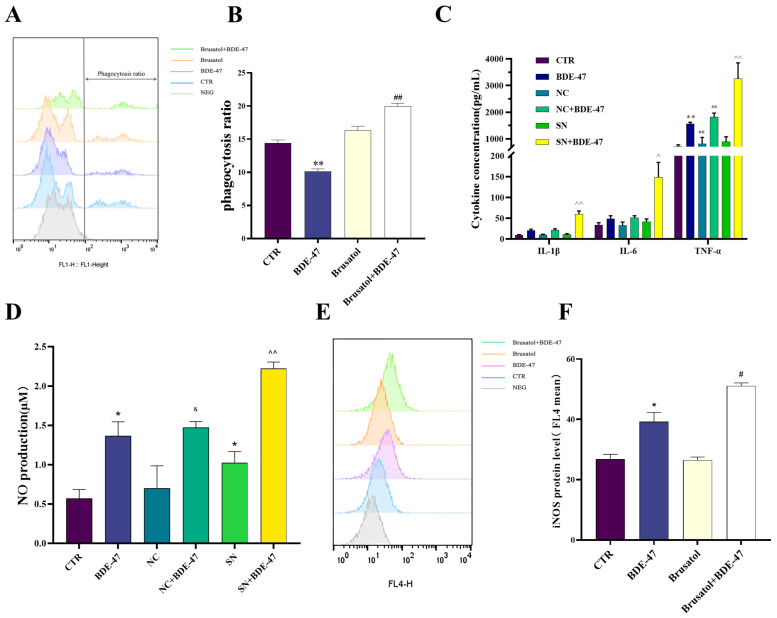
Effects of Nrf2 inhibition on macrophage immune function following BDE-47 exposure in RAW264.7 cells. (**A**,**B**) Phagocytic activity assessed by flow cytometry, including representative plots and quantitative analysis. (**C**) Secreted levels of pro-inflammatory cytokines IL-1β, IL-6, and TNF-α. (**D**) NO production. (**E**,**F**) Expression of iNOS, shown as flow cytometry plots and quantified median fluorescence intensity (MFI). Data are presented as mean ± SEM from three independent experiments (*n* = 3). Compared to the CTR group, * represents *p* < 0.05, ** represents *p* < 0.01. Compared to BDE-47, # represents *p* < 0.05, ## represents *p* < 0.01. Compared with NC, & represents *p* < 0.05 and && represents *p* < 0.01. Compared with NC+BDE-47, ^ represents *p* < 0.05 and ^^ represents *p* < 0.01.

BDE-47 exposure significantly reduced the phagocytic activity of RAW264.7 cells (Figure 7A,B). Treatment with the Nrf2 inhibitor brusatol alone showed a non-significant trend toward increased phagocytosis. Notably, co-treatment with BDE-47 and brusatol resulted in a significant increase in phagocytic activity compared with BDE-47 treatment alone (*p* < 0.01). The secretion of the pro-inflammatory cytokines, including IL-1β, IL-6 and TNF-α, was markedly increased in BDE-47-treated NC cells and SN cells (Figure 7C). Notably, cytokine levels in BDE-47-treated SN cells were significantly higher than those in BDE-47-treated NC cells (*p* < 0.01). Similarly, nitric oxide (NO) production was elevated in BDE-47-exposed groups, with the highest levels detected in BDE-47-treated SN cells (*p* < 0.01 vs. BDE-47-treated NC cells, Figure 7D). Inducible nitric oxide synthase (iNOS) expression was increased following BDE-47 treatment and was further enhanced by co-treatment with brusatol (Figure 7E,F). Collectively, these results indicate that Nrf2 suppression exacerbates BDE-47-induced inflammatory responses and impairs macrophage immune function.

## 4. Discussion

PBDEs, as persistent environmental pollutants, pose significant risks to higher organisms and human health due to their bioaccumulation and biomagnification along the food chain [44,45]. Among these compounds, BDE-47 is one of the most frequently detected congeners in environmental and biological samples and has been reported to exert neurotoxic, endocrine-disrupting, and developmental toxic effects, with increasing evidence of its immunomodulatory potential [14,46,47,48]. Our previous study demonstrated that BDE-47 induces intracellular ROS accumulation, mitochondria-dependent apoptosis, impaired phagocytosis, increased secretion of pro-inflammatory cytokines, and a tendency toward M1 polarization in RAW264.7 macrophages [23]. Although macrophages utilize ROS as part of their innate defense mechanisms, excessive ROS generation can lead to oxidative stress, macromolecular damage, and subsequent cell death and inflammatory dysregulation [49]. Despite these findings, current research on BDE-47-induced immunotoxicity remains largely descriptive, with limited understanding of the underlying molecular regulatory networks, particularly the cellular defense mechanisms activated in response to such stress. The nuclear factor erythroid 2-related factor 2 (Nrf2) signaling pathway is a key regulator of cellular antioxidant defense and has been shown to mitigate toxic effects induced by various environmental pollutants [31,50]. However, its specific role in BDE-47-induced macrophage immunotoxicity remains unclear. Therefore, the present study integrates network toxicology analysis with experimental validation to elucidate the molecular mechanisms underlying BDE-47-induced immunotoxicity in RAW264.7 macrophages, with a particular focus on the role of the Nrf2 signaling pathway.

### 4.1. Integrated Network Toxicology and Computational Analyses Identify Nrf2 as a Central Target in BDE-47-Induced Immunotoxicity

In this study, network toxicology analysis was performed to identify potential targets associated with BDE-47-induced immune dysfunction. Protein–protein interaction and modular analyses revealed subnetworks enriched in ROS-related biological processes, consistent with previous reports [26] and our previous findings [23]. Topological analysis based on degree and betweenness centrality further identified Nrf2 as a highly connected node within the oxidative stress-associated subnetwork. Although network-based approaches may be biased toward well-characterized genes, the prioritization of Nrf2 in this study is supported not only by its prominent topological features but also by its strong association with ROS-related pathways.

To further investigate this computational prediction, molecular docking and dynamics simulations were performed. Keap1 functions as a substrate adaptor of the Cullin3-RING ubiquitin ligase complex, maintaining Nrf2 in a suppressed state under basal conditions [51]. Certain xenobiotics have been reported to interact with Keap1, thereby modulating Nrf2 activity [52]. In the present study, molecular docking based on the crystal structure of the Keap1 Kelch domain suggested that BDE-47 may interact with the Keap1–Nrf2 binding interface, with a calculated binding energy indicative of moderate affinity. Moreover, molecular dynamic simulations indicated that the BDE-47–Keap1/Nrf2 complex remained relatively stable under the simulated conditions, as reflected by stable RMSD and limited RMSF. However, it should be emphasized that molecular docking and molecular dynamics simulations provide only theoretical estimations of ligand–protein interactions, and the predicted binding energy does not indicate high-affinity binding. Therefore, these findings should be interpreted as supportive evidence for a potential interaction rather than definitive proof. These computational observations are consistent with previous studies. For instance, isoginkgetin has been reported to exert antioxidant and cardioprotective effects through interaction with the Keap1–Nrf2 complex and subsequent activation of the Nrf2/ARE pathway [53]. In this context, our integrated computational analyses, together with experimental evidence of Nrf2 activation, suggest that BDE-47 may participate in regulation of the cellular antioxidant defense system, although the precise molecular mechanism requires further experimental validation. Importantly, our previous study demonstrated that BDE-47 induces a significant and dose-dependent increase in intracellular ROS, and that modulation of ROS levels markedly influences cellular injury [23]. These findings support the notion that oxidative stress represents a critical upstream event in BDE-47-induced toxicity.

### 4.2. BDE-47-Induced Activation of the Nrf2 Antioxidant Pathway

Nrf2 is a central regulator of cellular redox homeostasis and plays a key role in the adaptive response to oxidative stress [54]. Given that BDE-47 induces intracellular ROS accumulation, activation of the Nrf2 pathway is expected as a compensatory response. Under basal conditions, Nrf2 is sequestered in the cytoplasm through its interaction with Keap1, which targets it for proteasomal degradation. Oxidative or electrophilic stress disrupts this interaction, allowing Nrf2 to translocate into the nucleus and activate the expression of antioxidant and detoxifying genes [52]. In the present study, BDE-47 exposure triggered a dose-dependent activation of the Nrf2 pathway in RAW264.7 cells. No statistically significant difference in Nrf2 expression was observed between the untreated CTR and the DMSO vehicle control (*p* > 0.05), indicating that the solvent had no measurable effect. Western blot analysis showed that nuclear Nrf2 levels increased markedly at BDE-47 concentrations of 10 μM and above, accompanied by a corresponding decrease in cytoplasmic Nrf2, suggesting enhanced nuclear translocation. This effect became more pronounced at 40 μM, indicating a concentration-dependent activation of the Nrf2 pathway. Moreover, all BDE-47 treatment groups showed increases in nuclear Nrf2 accumulation when compared to both CTR and DMSO controls (*p* < 0.05 or *p* < 0.01), confirming that the observed Nrf2 activation is specifically attributable to BDE-47 exposure rather than solvent effects. Although nuclear Nrf2 levels remained elevated at higher concentrations, the response did not continue to increase proportionally, which may reflect the involvement of regulatory feedback mechanisms under conditions of excessive oxidative stress. Immunofluorescence analysis further confirmed the progressive nuclear accumulation of Nrf2 with increasing BDE-47 concentrations. Consistent with Nrf2 activation, qRT-PCR analysis showed upregulation of both classical oxidative stress-responsive genes (CAT, SOD1) and established Nrf2-dependent antioxidant targets (GCLC, GCLM, HO 1, NQO1). Notably, these genes exhibited distinct concentration–response patterns. While SOD1 and GCLC expression increased progressively up to 40 μM, CAT, HO-1 and NQO1 peaked at 20 μM and declined at higher concentrations, paralleling the biphasic pattern of nuclear Nrf2 accumulation. This divergence may reflect differential sensitivity of target genes to Nrf2 activity or the onset of cellular stress that partially impairs transcriptional responses at higher toxicant levels. These findings are consistent with the established role of Nrf2 in mitigating oxidative damage induced by various stressors, including H_2_O_2_ [55], haloacetates [56], cobalt chloride [57] and palmitic acid [58]. Collectively, these results demonstrate that BDE-47 activates the Nrf2 antioxidant defense system in a concentration-dependent manner. Importantly, this activation likely represents a primary adaptive response; the differential gene expression patterns and high-dose attenuation observed for specific targets hint at the complexity of cellular redox regulation and potential limitations of Nrf2-mediated protection under sustained or excessive BDE-47 exposure.

### 4.3. Nrf2 Deficiency Exacerbates BDE-47-Induced Cellular Damage and ROS Level

The protective role of Nrf2 in BDE-47-induced immunotoxicity was further investigated using both genetic and pharmacological inhibition approaches. Knockdown of Nrf2 via shRNA attenuated the BDE-47-induced upregulation of Nrf2 and its downstream targets and rendered macrophages more susceptible to BDE-47-induced injury. Cell viability assays showed that Nrf2-deficient cells exhibited a more pronounced reduction in viability following BDE-47 exposure, supporting a cytoprotective role for this pathway. Consistent with these findings, transmission electron microscopy revealed more severe ultrastructural alterations in Nrf2-suppressed cells after BDE-47 treatment, characterized by enhanced vacuolization and disrupted organelle integrity. Enlarged vacuoles containing electron-dense material were observed, suggesting increased cellular damage. These morphological changes were accompanied by a marked increase in intracellular ROS levels and a significant elevation in the apoptosis in Nrf2-deficient cells exposed to BDE-47. Together, these results indicate that Nrf2 contributes to limiting BDE-47-induced oxidative injury. Under physiological conditions, moderate ROS levels can activate the Nrf2 pathway, triggering a feedback response that enhances antioxidant defense and maintains redox homeostasis [59]. However, when Nrf2 function is impaired, this adaptive response is compromised. Consequently, BDE-47-induced ROS accumulation is insufficiently controlled, leading to enhanced cellular damage and increased apoptotic responses. Notably, although Nrf2 activation confers measurable protection, this effect appears to be partial and insufficient to fully counteract BDE-47-induced toxicity. Therefore, Nrf2 activation in this context likely represents an adaptive antioxidant response that mitigates, but does not completely prevent, cellular injury.

### 4.4. Nrf2 Regulates Macrophage Immune Function and Limits BDE-47-Induced Inflammatory Dysregulation

Beyond its canonical role in antioxidant defense, Nrf2 exerts broad regulatory effects on innate immunity, inflammatory responses, and macrophage polarization [60,61,62]. The present study provides functional evidence that Nrf2 modulates BDE-47-induced immune dysfunction in macrophages. Nrf2-deficient macrophages exposed to BDE-47 exhibited a dysregulated immune phenotype characterized by increased secretion of pro-inflammatory cytokines (IL 1β, IL 6, TNF α), elevated nitric oxide (NO) production, and enhanced expression of the M1 polarization marker iNOS. These findings suggest that Nrf2 normally acts to restrain excessive inflammatory activation under chemical stress. Interestingly, an apparent increase in phagocytic activity was observed in cells co-treated with BDE-47 and the Nrf2 inhibitor. This phenomenon likely reflects an aberrant activation state associated with enhanced inflammatory signaling. Pro-inflammatory (M1-like) macrophages often exhibit elevated phagocytic activity alongside increased cytokine production and nitric oxide synthesis. In this context, the enhanced phagocytosis observed under Nrf2 inhibition may represent a consequence of exacerbated inflammatory activation. Notably, this increase in phagocytic activity occurred concurrently with elevated ROS levels, enhanced apoptosis, and increased secretion of pro-inflammatory cytokines, indicating that macrophage function remained dysregulated. Therefore, these findings suggest that Nrf2 suppression promotes a hyperactivated but functionally imbalanced immune state. Collectively, these results indicate that Nrf2 functions as a critical immunomodulatory node that limits BDE-47-induced hyperinflammation responses and M1-like polarization, thereby contributing to the maintenance of immune homeostasis.

Although activation of the Nrf2 pathway is generally considered a protective response, it is also widely recognized as an adaptive mechanism triggered by oxidative stress. In the present study, BDE-47-induced Nrf2 activation is consistent with a compensatory response aimed at restoring cellular redox balance. Notably, functional inhibition of Nrf2 significantly aggravated BDE-47-induced cytotoxicity, inflammatory responses, and immune dysfunction, indicating that Nrf2 activation contributes to limiting cellular damage. However, this protective effect appears to be incomplete, as Nrf2 activation alone is insufficient to fully counteract BDE-47-induced toxicity. Taken together, these findings suggest that Nrf2 activation in response to BDE-47 exposure represents both an adaptive response to oxidative stress and a partially protective mechanism.

This study integrated network toxicology and computational analyses to identify Nrf2 as a central mediator of BDE-47-induced immunotoxicity. BDE-47 exposure activated the Nrf2 antioxidant pathway, while inhibition of Nrf2 exacerbated cytotoxicity, inflammatory cytokine production, and immune dysfunction, indicating that this signaling axis contributes to limiting cellular damage. These findings provide mechanistic insight into PBDE-induced immunotoxicity and highlight Nrf2 as a potential target for toxicological assessment and intervention strategies. However, several limitations should be acknowledged. The present study is based on the integration of in silico predictions with in vitro experiments conducted exclusively in the murine RAW264.7 macrophage line. Validation in additional models, such as human-derived THP-1 cells or primary macrophages, would strengthen the generalizability of these findings. Although molecular docking and molecular dynamics simulations suggested a potential interaction between BDE-47 and the Keap1–Nrf2 complex, direct experimental validation of this interaction is currently lacking. These computational approaches provide predictive and supportive insights rather than definitive proof of physical binding. In this study, activation of the Nrf2 pathway is supported by multiple complementary observations, including Nrf2 nuclear translocation, altered cytoplasmic–nuclear distribution, upregulation of downstream antioxidant genes, and functional changes following inhibitor co-treatment. Although transcriptional upregulation of Nrf2 downstream genes was observed, protein-level validation of these targets was not performed in the present study and warrants further investigation. Nevertheless, future studies employing co-immunoprecipitation, ubiquitination assays, and ARE-luciferase reporter assays are required to clarify the precise molecular mechanism involved. In addition, in vivo studies are needed to provide more physiologically relevant insights into the immunotoxic effects of BDE-47 and the protective role of Nrf2 under complex biological conditions. Such efforts will be essential for translating the present findings into environmentally relevant toxicological assessment and risk evaluation frameworks.

## 5. Conclusions

**Figure 8:**
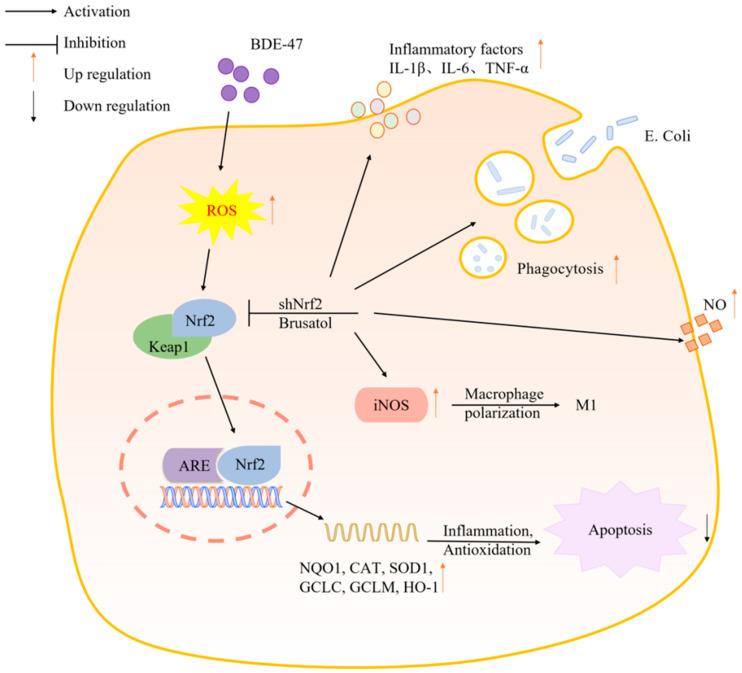
The mechanisms of the immunotoxicity induced by BDE-47 in RAW264.7 cells.

This study employed network toxicological analysis to identify Nrf2 as a key hub gene within the regulatory network of BDE-47-induced immunotoxicity, suggesting its central role. Molecular docking and molecular dynamics simulations further indicated that BDE-47 may interact with the Keap1–Nrf2 complex and form a relatively stable association under simulated conditions, providing supportive computational evidence for a potential interaction. Cellular experiments demonstrated that BDE-47 activates the Nrf2/ARE signaling pathway, as evidenced by Nrf2 nuclear translocation and the upregulation of downstream antioxidant genes, representing an adaptive cellular response to BDE-47-induced oxidative stress. Critically, inhibition of Nrf2 significantly enhanced the toxic effects of BDE-47. Nrf2-deficient cells exhibited more severe ultrastructural damage, elevated intracellular ROS levels, and enhanced apoptosis. Moreover, Nrf2 suppression aggravated BDE-47-induced macrophage immune dysfunction, as summarized in Figure 8. Collectively, these findings suggest that activation of the Nrf2/ARE signaling pathway contributes to mitigating BDE-47-induced cytotoxicity and plays a role in preserving macrophage immune function. However, this effect appears to be partial and reflects an adaptive cellular response to oxidative stress rather than a fully effective protective mechanism.

## Figures and Tables

**Table 1:** Specific primer sequences for real-time PCR.

Target Gene	Forward (5′-3′)	Reverse (5′-3′)
CAT	GCTCTCACATGGCTGCGAAGG	TCCTCAGGCTCGGCTTCACG
SOD	AACCAGTTGTGTTGTCAGGAC	CCACCATGTTTCTTAGAGTGAGG
Nrf2	AAGCACAGCCAGCACATTCTCC	TGACCAGGACTCACGGGAACTTC
GCLC	CCATTACCACCTGCTGTCTG	GCCTGTCAATCTGCTCCTG
GCLM	CTTCGCCTCCGATTGAAGATG	AAAGGCAGTCAAATCTGGTGG
HO-1	ACCGCCTTCCTGCTCAACATTG	CTCTGACGAAGTGACGCCATCTG
NQO1	GCCGAACACAAGAAGCTGGAAG	GGCAAATCCTGCTACGAGCACT
GAPDH	CATCACTGCCACCCAGAAGACTG	ATGCCAGTGAGCTTCCCGTTCAG

## Data Availability

The data presented in this study are available from the corresponding author upon reasonable request.

## Supplementary Materials

The following supporting information can be downloaded at: https://www.mdpi.com/article/10.3390/biom16050674/s1, File S1: Western Blotting Figure.
