# Supplementary material for: Activation of the Nrf2/ARE Pathway Attenuates BDE-47-Induced Immunotoxicity in RAW264.7 Macrophages
Source: Biomolecules. 2026 May 1;16(5):674. doi: 10.3390/biom16050674 (PMC13204577; doi:10.3390/biom16050674)
Supplement: Supplementary file 1 [file biomolecules-16-00674-s001.zip › biomolecules-4235931-supplementary.pdf]

Original Images for Blots (Treatment with different concentrations of BDE-47)

**Nucleus-Nrf2**	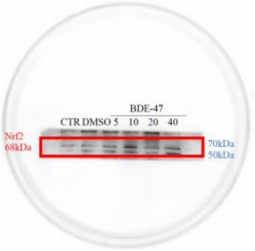	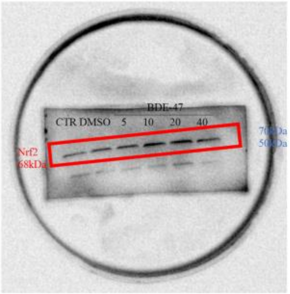<br>Used in the manuscript	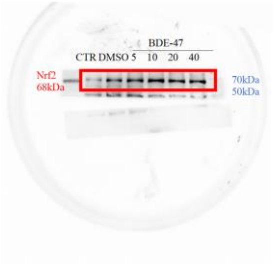
**Histone H3**	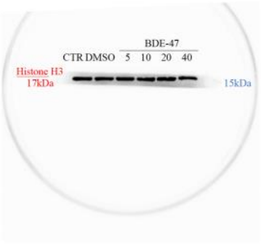	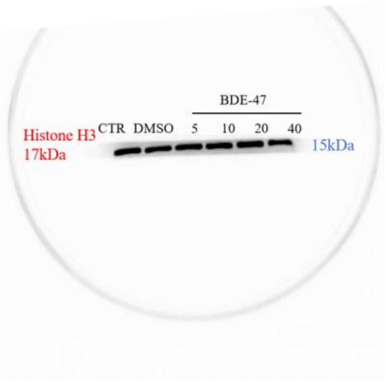<br>Used in the manuscript	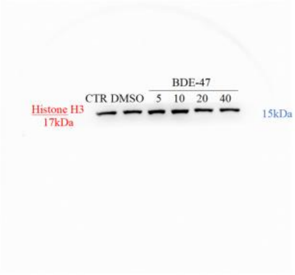
**Cytoplasm-Nrf2**	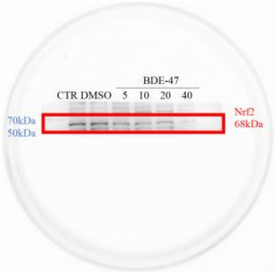	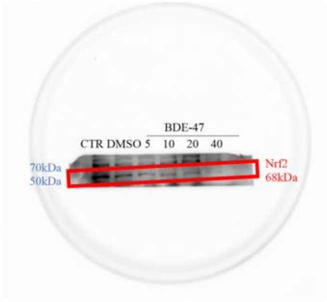<br>Used in the manuscript	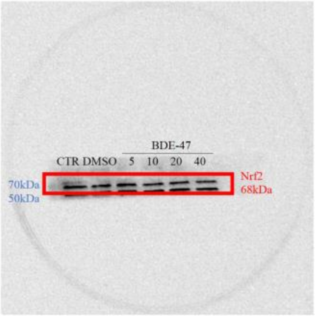
**β-actin**	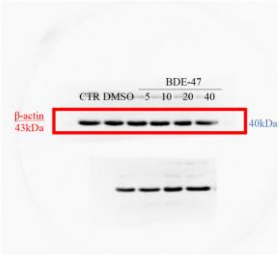	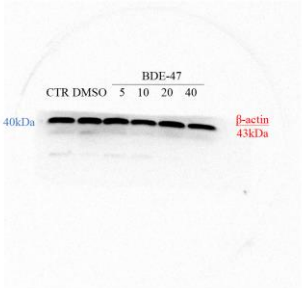<br>Used in the manuscript	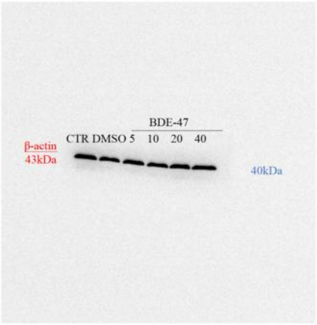

Sample loading order (from left to right): CTR, DMSO, 5μM, 10μM, 20μM, 40μM
